# Bilateral maculopathy following electrical burn: case report

**DOI:** 10.1590/1516-3180-2014-1326643

**Published:** 2014-08-22

**Authors:** Leandro Dario Faustino, Ramon Antunes Oliveira, Andrea Fernandes Oliveira, Eduardo Büchelle Rodrigues, Nilva Simeren Diego Moraes, Lydia Masako Ferreira

**Affiliations:** I MD. Resident Physician in Plastic Surgery, Universidade Federal de São Paulo (Unifesp), São Paulo, Brazil; II MD. Resident Physician in Ophthalmology, Universidade Federal de São Paulo (Unifesp), São Paulo, Brazil; III MD, MSc. Coordinator of Burn Care Unit, Plastic Surgery Division, Department of Surgery, Universidade Federal de São Paulo (Unifesp), São Paulo, Brazil; IV MD, PhD. Ophthalmology Professor, Department of Ophthalmology, Universidade Federal de São Paulo (Unifesp), São Paulo, Brazil; V MD, PhD. Associate Professor, Department of Ophthalmology, Universidade Federal de São Paulo (Unifesp), São Paulo, Brazil; VI MD, PhD. Chairwoman and Full Professor in the Plastic Surgery Division, Department of Surgery, Universidade Federal de São Paulo (Unifesp), São Paulo, Brazil

**Keywords:** Burns, Burns, electric, Retina, Eye burns, Retinal detachment, Queimaduras, Queimaduras por corrente elétrica, Retina, Queimaduras oculares, Descolamento retiniano

## Abstract

**CONTEXT:**

Electrical burns are an important etiology in dealing with patients suffering from burns. In situations of extensive deep lesions of multiple organs and systems affecting young and economically active people, there is a need for expensive multidisciplinary treatment, with a high socioeconomic cost for the community. Among the permanent injuries that explain this high cost, eye injuries stand out, since they are widely disabling. Although rare, lesions of the posterior segment of the eye are associated with higher incidence of major sequelae, and thus deserve special attention for dissemination and discussion of the few cases observed.

**CASE REPORT:**

The authors report the case of a patient who suffered high-voltage electrical burns and presented bilateral maculopathy, which evolved with a need for a surgical approach to repair retinal detachment and permanent low visual acuity.

**CONCLUSION:**

This report highlights the rarity of the etiology of maculopathy and the need for campaigns for prevention not only of burns in general, but also especially of electrical burns.

## Abstract (pt)

**CONTEXTO:**

As queimaduras elétricas representam etiologia importante na abordagem de pacientes vítimas de queimaduras. Lesões extensas e profundas de múltiplos órgãos e sistemas e o acometimento de população jovem economicamente ativa apontam para a necessidade de tratamento multidisciplinar dispendioso, com alto custo socioeconômico para as comunidades. Dentre as lesões permanentes que justificam esse alto custo, as oculares têm local de destaque, porque são amplamente incapacitantes. Embora raras, as lesões do segmento posterior do olho estão associadas a maior incidência de sequelas importantes, merecendo atenção especial para divulgação e discussão dos poucos casos observados.

**RELATO DE CASO:**

Os autores relatam o caso de paciente vítima de queimadura elétrica com fio de alta voltagem que apresentou maculopatia bilateral, evoluindo com necessidade de abordagem cirúrgica para correção de descolamento de retina e baixa acuidade visual permanente.

**CONCLUSÃO:**

Este relato destaca a raridade da etiologia da maculopatia e a necessidade de campanhas de prevenção não só das queimaduras em geral, mas especialmente das queimaduras elétricas.

## INTRODUCTION

Electrical burns occur when human tissues contact a voltaic arc created by an electric potential difference.1 The resulting lesions may be secondary to the direct passage of electric current through the human body or may merely be due to the individual's prox imity to the arc created, in which case the current passes through other media, such as the air and conductive objects.^1^^,^ 2

The estimated incidence of electrical burns ranges from 5% to 20% among all causes of burns.^2^ Thus, they are an important etiology of burns in the general population, not only because of the absolute number of cases, but also because they are often associated with severe and deep injuries, high treatment costs and prolonged hospitalization.^3^^,^ 4 In a retrospective study on more than 800 burn victims, Luz et al. reported a mean length of hospital stay of 34 days, with more than 120 surgical procedures performed over a period of five years, including skin grafts, amputations, escharotomy and debridement.^3^ These epidemiological characteristics are closely related to the main types of energy sources, divided into high (> 1,000 V) and low (< 1,000 V) energy sources.^2^^,^ 3 The voltage difference between two points is the main determinant of the amount of electricity that will move between them, as postulated by Ohm's Law, which states that electric current and voltage are directly proportional variables, depending on the resistance of the conductive material.^1^ In the human body, the skin has the highest estimated resistance among tissues (approximately 100,000 Ω),^5^ which results in smaller quantities of electricity on the skin. Deep tissues such as subcutaneous cellular tissue, muscle and bones, with lower resistance (estimated total resistance of 300 Ω for the set of internal human tissues),^5^ usually allow higher currents, which explains the extensive deep lesions found in this type of burn. 

Besides the magnitude of electron flow, the effects of current in the human body also depend on the pathway followed, duration of contact with the energy source and mechanisms of contact.^1^^,^ 5 Lesions may be caused by direct contact between the human body and an ionized surface (the most common mechanism); by indirect transmission of an electric current through a conducting medium close to the patient such as air or water (voltaic arc); by combustion and fire originating in gases and clothes near the victim ("flash burns"); and by direct conduction of electric current through human tissues.^6^

The interaction between electric current and different tissues takes place through direct cell injury or conversion of electricity into heat. The sudden cell membrane depolarization caused by the electric shock gives rise to cell dysfunction and death, and the dissipated heat causes severe burns not only at the entry and exit points, but also all along the path of the electric current. Increased intracompartmental pressure is a direct consequence of these cellular mechanisms of electrical injuries, thus explaining the high rates of limb losses and fasciotomy observed in this type of burn.^2^^,^ 3 ^,^ 7 It has been estimated that while currents below 1 mA are often imperceptible, currents of 20 mA may lead to paralysis of respiratory muscles, sometimes progressing to ventricular fibrillation if the amperage reaches 100 mA.^5^ Contact with high-voltage sources either directly or through a voltaic arc has been widely correlated with severe burns, with predominance of third-degree skin injuries, extensive destruction of deep tissue and infectious complications during hospital stay.^3^ Burns of "flash burn" nature have been associated with large areas of body injuries, with predominance of second-degree injuries.^3^^,^ 5

Here, the case of a patient who suffered high-voltage electrical burns and presented bilateral maculopathy, with evolution requiring a surgical approach in order to repair retinal detachment and permanent vision loss, is reported along with a brief review of the subject in the specialized literature.

## CASE REPORT

The patient was a 40-year-old male bricklayer, who was brought to the hospital as a victim of electrical shock at his workplace caused by a high-voltage wire of the municipal power grid. The patient presented with airway patency and spontaneous breath ing; he was hemodynamically stable, oriented and alert, with third-degree burns in the abdomen (entry wound) and feet (exit wounds) and second-degree burns in the right frontal region (2% of total body surface affected), with associated bilateral perior bital edema. He reported a severe vision loss in both eyes asso ciated with marked photophobia. The patient denied having any previous ophthalmic surgery or illness. 

The patient was admitted to the Burn Care Unit of Hospital São Paulo, (Department of Surgery, Plastic Surgery Division, Universidade Federal de São Paulo, Unifesp, São Paulo, SP, Brazil). He was treated with daily dressings containing 1% silver sulfadiazine and received intensive support from a multidisciplinary team (pain team, infectologists, ophthalmologists, specialized nurses, psychologists and physiotherapists).

**Figure 1:**
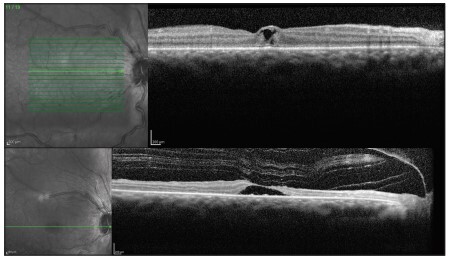
Optical coherence tomography showing intraretinal macular cysts in the right eye.

**Figure 2:**
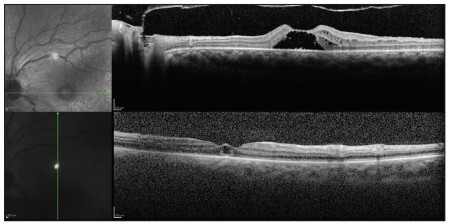
Optical coherence tomography showing intraretinal macular cysts in the left eye.

An ophthalmic examination carried out three days after the electric shock revealed poor visual acuity of hand movements, which was only enough to detect hand movements in the right eye and to count fingers at 50 centimeters distance from the left eye, with conjunctival chemosis, mild corneal edema with diffuse punctate keratitis, transparent lens and no changes in pupil reflexes, intraocular pressure or retinal mapping. He was treated with pomade containing 10,000 IU of retinol acetate and artificial tears, and underwent optical coherence tomography (OCT). The examination revealed intraretinal macular cysts in both eyes, to the inner nuclear layer (Figures 1 and 2). 

**Figure 3:**
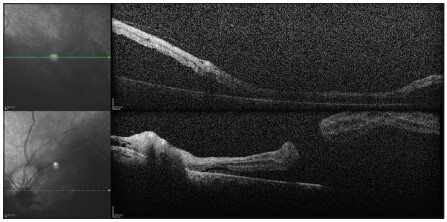
Optical coherence tomography showing bilateral retinal detachment.

The patient progressed with satisfactory healing of burn areas, without the need for surgical debridement or grafting. He was discharged 12 days after admission and was followed up in the Burns Outpatient Clinic and Retina Outpatient Clinic. At the 60-day return to the ophthalmic service, the patient presented rhegmatogenous retinal detachment with macular holes in both eyes, affecting the maculae and sparing only the upper third of the retina, without peripheral tears (Figure 3). The patient underwent retinal surgery (*pars plana* vitrectomy) in both eyes with an interval of 15 days between the eyes, without complications. His condition progressed during the postoperative period with lens opacification in both eyes and new retinal detachment in the right eye, which was again submitted to cataract (phacoemulsification with intraocular lens implantation) and retinal surgery (*pars plana* vitrectomy with silicone oil). No tears were observed.

By the time of submitting this manuscript, almost 120 days after the electric shock, the patient was progressing with a good healing process in the skin areas affected by the electric current. However, his visual prognosis was poor despite the procedures performed by the ophthalmic monitoring team. His visual acuity comprised perception of light in his right eye and hand motion in his left eye, with transparent cornea, intraocular lens and attached retina.

## DISCUSSION

Electrical burns are considered to be particularly important because the resulting lesions are secondary to the direct passage of electric current through the person.^1^^,^ 2

The portion of the population most affected by electrical burns has been classically described as young male adults (19-50 years of age). They suffer these burns in their workplaces, mainly in industrial, construction and mining activities,^8^ the sectors in which high-voltage electrical equipment is concentrated. Unintended manipulations of transmission lines for illegal access to the power grid have also been the cause of electrical burns in developing countries.^7^ Domestic accidents account for a small fraction of cases, with a different epidemiological spectrum and less severe injuries (low voltage) in pediatric patients (< 10 years of age) and females.^9^ Our case of a 40-year-old male bricklayer who was injured at his workplace through an electric shock from a high voltage wire is completely in line with the literature. 

With regard to specific lesions of different systems and human organs associated with electrical burns, there is a broad spectrum of clinical forms. The overall mortality rate associated with high-voltage sources has been described as approximately 11%.^3^ Amputations and dysesthesia associated with peripheral nerve injuries are the main forms of injury to the extremities, showing variable incidence according to the sources (3% in the United States, 3-5% in China, 10% in Brazil, 3-9% in India and 16% in Turkey).^7^ Myoglobinuria and acute renal failure due to large muscle destruction are common complications in burn units. Cardiac dysfunction, such as atrial or supraventricular fibrillation can also be observed, as well as recurrent pericarditis. Cerebral infarction, hypoxic encephalopathy and plegia of ischemic origin have been correlated with the reactive vasospasm that has been described in victims of major electric discharges.^10^ Cognitive and psychological deficits may be disabling after electrical burns. Also, otological disorders such as deafness, dizziness, conduction defects, fractures of the mastoid and auditory ossicles have already been described as consequences of electric shock.^10^

Ophthalmic injuries have also been reported in patients suffering from electrical burns. More than half of all lightning victims present some kind of ophthalmic injury, mostly involving the anterior segment of the eye.^11^ Burns of the eyelids, thermal keratitis and presence of hyphema are the most frequent initial lesions observed in these patients. Later, with protein degeneration and decreased permeability of the cornea due to passage of electric current, electrical cataract occurs, usually bilaterally.^12^ In this type of injury, both the anterior and the posterior lens may be affected, and the clinical scenario is quite variable, with the possibility of partial or even total regression of opacity.^13^ Uveitis induced by electric shock (less than ten cases described in the literature) and ankyloblepharon secondary to electrical trauma (one reported case) are rare lesions of the anterior segment of the eye (Table 1).^14^

**Table 1:** Search strategies implemented on May 4, 2013, and results from Medline, Lilacs (Literatura Latino Americana e do Caribe em Ciências da Saúde) and Embase

Database	Search terms	Results	Relevant findings
Medline (via PubMed)	((“burns, electric” [MeSH Terms]) OR (“burns, eye” [MeSH Terms])) AND ((“retinal detachment” [MeSH Terms]) OR (“retinal perforations” [MeSH Terms]))	5 studies	Macular holes and cysts are commonly described associated with little or no retinal detachment
Lilacs (via Bireme)	Burns, Electric [Subject descriptor] and Eye Burns [Subject descriptor] and Retinal Detachment [Subject descriptor]	0 articles	There were no articles or case reports found in any language
	Queimaduras por Corrente Elétrica [DeCS Category] and Queimaduras Oculares [DeCS Category] and Descolamento retiniano [DeCS Category]	0 articles	
	Quemaduras por Electricidad [DeCS Category] and Quemaduras Oculares [DeCS Category] and Desprendimiento de Retina [DeCS Category]	0 articles	
Embase (via Elsevier)	burn’/exp AND ‘electric burn’/exp AND ‘retina detachment’/exp	0 articles	None

Neurological lesions and lesions in the posterior segment of the eye are less frequent. Thermal papillitis, loss of pupil response, Horner's syndrome and unilateral or bilateral neuropathy have been reported,^13^ including associated scenarios of blindness. The maculae have been described as a region that is particularly sensitive to thermal damage because of its high concentration of melanocytic granules, which increases the resistance and leads to greater heat dissipation when struck by electric current.^14^ Local inflammation seems to contribute towards retinal pigment epithelium dysfunction, thereby causing intraretinal edema that has been described as cysts or macular holes. It has also been postulated that local ischemia and vitreomacular traction contribute towards this mechanism.^15^^,^ 16 During the progression of these cases, retinal thickening secondary to cystic macular changes may regress without intervention, while rare and disabling complications such as retinal detachment may require surgical procedures to prevent sequelae. This kind of complication is rare and, although etiologically different, it seems to progress in a way similar to the scenarios of traumatic or idiopathic retinal detachment.^16^^-^ 18

Retinal lesions associated with high-voltage shock such as from exposed wires and the power grid are even rarer than those described in lightning injuries, and they include macular cysts,^14^^,^ 19 macular holes and retinal detachment.^20^^,^ 21 OCT is a supplementary examination that is fundamental for distinguishing between cysts and partial or full-thickness lamellar holes.^22^ However, full-thickness macular holes have not been found to naturally progress to retinal detachment, unless there are peripheral tears. In the case reported here, it is postulated that retinal detachment may have occurred secondary to the full-thickness macular hole, or to peripheral microtears that were not identified at surgery. There have been reports of a stippling pattern in retinal pigment epithelium (RPE) in fluorescein angiography examinations on a patient after lightning shock, which also suggests that there was involvement of the peripheral retina in this etiology of trauma.^12^

## CONCLUSIONS

The possible complications and sequelae of ophthalmic inju ries include permanently decreased visual acuity and blind ness. These, together with injuries to other organs and systems and the epidemiological characteristics of electrical trauma, affecting a young and economically active population through disabling sequelae and prolonged hospital stay, reflect the importance of electrical burns within a broader population based context that goes beyond the care from a burns special ist. The multiple lesions found in these patients show the need for a standardized multidisciplinary approach, inside and out side the hospital, which, for the public health system, can be highly expensive. 

As well as traumatic injuries in general, prevention through public awareness campaigns and close monitoring of working conditions in industrial sectors using large power grid networks seems to be the most cost-effective approach towards burns patients, especially in relation to electrical burns. 
